# Heterogenic Genetic Background of Distal Arthrogryposis—Review of the Literature and Case Report

**DOI:** 10.3390/children11070861

**Published:** 2024-07-16

**Authors:** Anett Illés, Henriett Pikó, Virág Bartek, Olívia Szepesi, Gábor Rudas, Zsófia Benkő, Ágnes Harmath, János Pál Kósa, Artúr Beke

**Affiliations:** 1 Department of Internal Medicine and Oncology, Semmelweis University, 1085 Budapest, Hungary; illes.anett@semmelweis.hu (A.I.); piko.henriett@semmelweis.hu (H.P.); kosa.janos@semmelweis.hu (J.P.K.); 2 Department of Obstetrics and Gynecology, Semmelweis University, 1085 Budapest, Hungary; virag.bartek@semmelweis.hu (V.B.); szepesi.olivia@semmelweis.hu (O.S.); benko.zsofia@semmelweis.hu (Z.B.); harmath.agnes@semmelweis.hu (Á.H.); 3 Heim Pál National Pediatric Institute, 1085 Budapest, Hungary; rudas.gabor@semmelweis.hu

**Keywords:** distal arthrogryposis, genetic variants, review of the literature

## Abstract

Distal arthrogryposis (DA) is a skeletal muscle disorder that is characterized by the presence of joint contractures in various parts of the body, particularly in the distal extremities. In this study, after a systematic review of the literature, we present a case report of a non-consanguineous family. In our case, the first-trimester ultrasound was negative, and the presence of the affected mother was not enough for the parents to consent to us performing invasive amniotic fluid sampling. The second-trimester ultrasound showed clear abnormalities suggestive of arthrogryposis. Whole-exome sequencing was performed and an autosomal dominantly inherited disease-associated gene was identified. In our case, a pathogenic variant in the TNNT3 gene c.188G>A, p.Arg63His variant was identified. The mother, who had bilateral clubfoot and hand involvement in childhood, carried the same variant. The TNNT3 gene is associated with distal arthrogryposis type 2B2, which is characterized by congenital contractures of the distal limb joints and facial dysmorphism. In the ultrasound, prominent clubfoot was identified, and the mother, who also carried the same mutation, had undergone surgeries to correct the clubfoot, but facial dysmorphism was not detected. Our study highlights the importance of proper genetic counseling, especially in an affected parent(s), and close follow-up during pregnancy.

## 1. Introduction

### 1.1. Epidemiology, Characteristics, and Genetics

About 1 in 3000 live births presents with some form of arthrogryposis, many of which are nonprogressive and improve with physiotherapy, but extremely severe and lethal forms also exist [1]. 

The pathomechanism in all cases is a lack of fetal movement due to intrinsic or extrinsic causes [2,3]. Fetal joints begin to form in the 5th and 6th gestational weeks, and adequate fetal movement is essential for their proper development [4]. A lack of movement causes connective tissue to accumulate in the joints, which causes them to become stiff and then contract. The etiology may include genetic causes, congenital infections, external factors that impede the movement of the uterus (e.g., oligohydramnios, fibrotic uterus, Simonart’s ligament), or maternal autoimmune diseases (e.g., Myasthenia gravis) [5]. Among the teratogenic agents that affect the fetus in the first trimester, curare, misoprostol, cocaine, and alcohol may contribute to the development of arthrogryposis [6].

The currently accepted classification of arthrogryposis is as follows [6].

1. Arthrogryposis with normal neurological function.

- Amyoplasia: The symmetric absence of muscle development, mostly in the limbs. The etiology is unclear. The upper limb is rotated at birth with an extended elbow and a flexed wrist. Clubfoot of varying severity is seen. Associated anomalies include frontal hemangioma and genital and finger hypoplasia. Gastroschisis is present in 10% of cases. Affected children have normal intelligence and no craniofacial abnormalities.
- Distal-type arthrogryposis (DA): An autosomal dominant inherited disorder affecting mainly the lower limbs, excluding the large joints. No clear neuro- or myogenic abnormalities are underlying. The upper limb is affected to a lesser extent, with ulnar deviation in the form of camptodactyly. The phenotype is highly heterogeneous.
- Systemic connective tissue diseases: Contractural arachnodactyly, Beals syndrome, Larsen syndrome, and Ehlers–Danlos syndrome. Ligamentous disorders lead to restricted joint motion.

c/1 Beals syndrome: The inheritance of this syndrome is autosomal dominant, characterized by contractures, camptodactyly, arachnodactyly, scoliosis, and hypoplasia. The most commonly affected gene is FBN2 [7].
c/2 Larsen syndrome: The inheritance of this syndrome is autosomal dominant or sporadic, characterized by joint dislocation, spine anomalies, and characteristic facial features. The most commonly affected gene is FLNB [8].
c/3 Ehlers–Danlos syndrome: This is a heterogenous disease with 14 subtypes. Common features include joint hypermobility, skin abnormalities, and wound-healing problems. Arthrogryposis can happen but is not present in all cases [9].

d. “Multiple pterygium” syndrome: There are lethal and non-lethal (Escobar-type) forms of this syndrome. Its genetics and phenotypic presentation are highly heterogeneous.
  - Lethal form: This form involves IUGR/SGR (intrauterine growth retardation/small for gestational age), contractures, pterygia, and a dysmorphic face. Affected individuals are stillborn or do not survive the perinatal period. Camptodactyly may occur with or without syndactyly. Minor facial anomalies, ptosis, and antimongoloid eyes are often seen.
  - Non-lethal/Escobar type: This form involves scoliosis, cryptorchidism, and facial involvement with normal intelligence
e. Oligohydramnios/external factors

2. Arthrogryposis with neurological involvement: This is caused by teratogenic agents, intrauterine infections, or aneuploid chromosomes. Neural migration is involved, resulting in cerebral or cerebellar hypoplasia, or holoprosencephaly, but other neural structures may also be affected. Werdnig–Hoffmann syndrome and spinal muscular atrophy are the most common forms. In addition to contractured joints, affected individuals have low intelligence, epilepsy, and severe learning difficulties.

Wahling et al., in a retrospective study of 417 patients, found that proportionally, 25.7% of their studied patients suffered from amyoplasia, 18% from distal-type arthrogryposis, and 56.4% from other types of arthrogryposis [10].

As of 2022, 402 genes [11] have been identified in the background of arthrogryposis [11,12]. Ghaoui et al. identified a genetic mutation in 45% of their investigated cases (n = 60 families) [13]. Laquerriere et al. identified a genetic mutation in 52.7% of their investigated cases (n = 315 families) [11].

The ten most common gene variants and their mechanisms of action found by Laquerriere et al. in a cohort analysis of 315 families are described in Table 1 [11].

As can be seen, the most common mutations affect the genes encoding proteins that affect muscle contraction. Mutations in the TTN, CHRNG, RYR1, and ECEL1 genes were detected in 46 (27.7%) of the 166 families studied [11].

Other genes involved include genes encoding proteins involved in the formation of the Ranvier junction (CNTNAP1, GLDN), genes encoding proteins involved in peripheral myelination (ADCY6, ADGRG6, LGI4), and genes encoding proteins involved in brain development (MAGEL2) [11]. Year after year, new gene mutations are identified in the background of arthrogryposis. In addition to de novo, sporadic cases, AD, AR, and sex-linked inherited forms are known. As expected, the most common mode of inheritance is autosomal recessive [23]. 

### 1.2. Ultrasound Findings

In prenatal screening, arthrogryposis is indicated by the absence of fetal movement, i.e., fetal akinesia. If suspected, it is recommended to scan all limb regions for at least 45 min. The severity of arthrogryposis is in direct proportion to the degree of absence of fetal movement. In high-risk pregnant women with a positive history, it is recommended that fetal movements are also examined at 14, 16, 18, 20, and 22 weeks [6].

Specific ultrasound findings in arthrogryposis include flexion abnormalities in both the proximal and distal joints. In addition to the extremities, the jaw, spine, and cervical region may also be affected. Hypomineralization of the long bones may also be detected. 

Contractures are usually diagnosed in the second trimester, but reduced fetal movement is seen as early as the eighth week [6]. Cystic hygroma may be present in the first trimester. If associated with cystic hygroma, the disease is often lethal and intrauterine. 

Associated abnormalities may affect the craniospinal system. Cerebral or cerebellar hypoplasia, ventriculomegaly, and holoprosencephaly may develop depending on the underlying cause. If arthrogryposis is part of primary myopathy, it may be associated with gastroschisis and intestinal atresia. 

Polyhydramnios can develop due to reduced amniotic fluid absorption, but oligohydramnios can contribute to the development of arthrogryposis, even as a causal factor, so amniotic fluid abnormalities are not specific. Patients with the Pena–Shokeir phenotype may present with micrognathia, cleft palate, hypertelorism, IUGR/SGR, or pulmonary hypoplasia [1]. A 3D ultrasound and fetal MRI may be helpful to examine the joints. 

The literature suggests that arthrogryposis is only diagnosed prenatally in 25% of pregnancies, usually in more severe cases [24]. This may be because fetal limb movement testing is time-consuming, requires an experienced examiner, and is particularly difficult in the first trimester. 

### 1.3. Antenatal Care

In the case of arthrogryposis, a detailed ultrasound examination and targeted fetal cranial or cardiac ultrasound should be performed. A fetal MRI should be considered to clarify the diagnosis. A maternal TORCH scan may be recommended. 

If the diagnosis is confirmed, close neonatological, pediatric neurological, or pediatric orthopedic care is required from the time of diagnosis. Termination of pregnancy may be offered based on the genetic indication. Termination of delivery at a tertiary center in a hospital with a PIC background is recommended. Due to jaw discrepancies, we must be prepared for a difficult airway. Osteoporotic deviations predispose patients to traumatic fractures during childbirth [1].

### 1.4. Prognosis and Treatment

The prognosis depends largely on the underlying causes, genetic factors, and the presence of associated intrauterine infections. In milder cases, contractures may improve with pediatric orthopedic care and physiotherapy [1]. Surgical treatment includes tendon reconstruction, extensor lengthening, repositioning, and cast fixation. In scoliosis, spinal stabilization procedures are necessary. A difficult airway can be relieved by mandibular reconstruction, which requires a multidisciplinary surgical team. Surgical reconstructive surgery and rehabilitation with a trained physiotherapist should be started as soon as possible, but the recurrence of deformities is common and may require further intervention [24].

If surgical reconstruction is successful, psychosocial disadvantages are present in affected patients due to ongoing pain, fatigue, and mobility difficulties [25].

In the clinical practice, supportive care for patients is offered to improve their quality and quantity of life toward independent living; in this setting, occupational therapy, physical therapy, and surgery can be applied [26].

Although these treatments could improve both patients’ and their families’ lives, further investigations are needed to understand the connection between genetic variants and disease manifestation, which eventually leads us to identify the most effective therapy for them. With a proper understanding of the genotype–phenotype correlation, family management could be further supported both in prenatal and preimplantation settings.

Despite the continuously evolving knowledge about the molecular etiology of arthrogryposis, the potential genetic background behind the symptoms remains unclear in many cases. Due to the complexity of the disease, the potential genetic architecture and underlying genetic etiology of certain cases with arthrogryposis are identified only through the high-throughput screening of coding regions. Additionally, the expanding number of related genes and several other syndromes that could have symptoms similar to distal arthrogryposis highlight the necessity of whole-exome sequencing. Both the mono- and biallelic variants of several genes, responsible for autosomal dominant (AD) and autosomal recessive (AR) disease, respectively, are recognized to result in either the same or a different syndrome and are identified more frequently with whole-exome sequencing. Recently, potential digenic or oligogenic inheritance in cases of diseases arose [27].

### 1.5. Distal Arthrogryposis (DA)

In our case report, we describe a case with distal arthrogryposis, so hereby, we discuss the relevant literature.

Distal arthrogryposis (DA) is a skeletal muscle disorder characterized by multiple congenital joint contractures present at birth. [3]. In distal arthrogryposis, joint contractures affect various parts of the body, particularly in the distal extremities, including the hands, wrists, ankles, and feet. 

In clinical practice, the manifestations in the lower extremities commonly include clubfoot and vertical talus, while the proximal joints are largely spared, in the absence of primary neurologic and/or muscle disease affecting limb function. Currently, distal arthrogryposis is classified into more than 10 types, and this number is continuously growing due to the use of high-throughput molecular genetic techniques (Table 2). Myopathy is one of the most commonly manifested phenotypes, but the diagnostic features of DA also include camptodactyly or pseudo-camptodactyly, hypoplastic or absent flexion creases, overriding fingers, ulnar deviation at the wrist, talipes equinovarus, calcaneovalgus deformities, vertical talus, and/or metatarsus varus [26].

Currently, 12 types of distal arthrogryposis are distinguished, which are discussed in Table 2 [28].

**Table 2:** Classification of distal arthrogryposis and the associated genes.

Classification	Clinical Features	Gene	Inheritance
Distal arthrogryposis type 1	Camptodactyly and clubfoot	*TPM2*	AD
Distal arthrogryposis type 2A (Freeman–Sheldon syndrome)	Contractures of fingers and toes, kyphosis, scoliosis, and whistling face	*MYH3*	AD
Distal arthrogryposis type 2B (Sheldon-Hall syndrome)	Features similar to distal type 1 and type 2A with distal joint contractures in the limbs, triangular face, downward-slanting palpebral fissures, small mouth, and high-arched palate	*TNNI2, TNNT3, MYH3, TMP2*	AD
Distal arthrogryposis type 3 (Gordone syndrome)	Short stature, cleft palate, and palatoschisis	*PIEZO2*	AD
Distal arthrogryposis type 4	Contractures and severe scoliosis	N/A	AD
Distal arthrogryposis type 5	Limitation of eye movement (opthalmoplegia), ptosis and strabismus	*PIEZO2*	AD
Distal arthrogryposis type 6	Sensorineural hearing loss	N/A	N/A
Distal arthrogryposis type 7	Trismus-pseudocamptodactyly, short stature, and shortened paralyzed muscles	*MYH8*	AD
Distal arthrogryposis type 8	Autosomal dominant multiple pterygium syndrome	*MYH3*	AD
Distal arthrogryposis type 9	Congenital contractural arachnodactyly, phenotypes similar to those of Marfan syndrome, but without cardiovascular abnormalities	*FBN2*	AD
Distal arthrogryposis type 10	Congenital plantar contractures	2q	AD
Distal arthrogryposis type 11	Camptodactyly, absent flexion creases, and limited forearm supination	*MET*	AD
Distal arthrogryposis type 12	Congenital contractures (small joints of the fingers and toes), contractures of the knees and Achilles tendons, spinal stiffness, scoliosis, and orthodontic abnormalities	*ADAMTS15*	AR

The information in this information is based on the OMIM database (8 May 2024). Abbreviations: AD—autosomal dominant; AR—autosomal recessive; N/A—no data available [29].

Based on the literature reviewed, a brief description of the most common subtypes follows.

Distal arthrogryposis type 1. The main characteristic of this subtype is joint deformities (contractures), mainly in the hand and foot. It is characterized by camptodactyly, overlapping fingers, and deformity of the hand (abnormal digital flexion, as well as ulnar deviation). Clubfoot is common. The phenotypes vary from individual to individual, but other organ systems are usually not affected. [30] The mode of inheritance is autosomal dominant, with TNNI2, TNNT3, TPM2, MYH3, and MYBPC1 [31] being the most commonly affected genes [32]. TNNI2, TNNT3, and TPM2 can cause DA2B phenotypes in some cases [32]. 

Distal arthrogryposis type 2B. The features of DA2B, also known as Sheldon–Hall syndrome, in addition to the characteristics described in type 1, tend to have characteristic facial features (oral abnormalities, down-slanting palpebral fissures, micrognathia). Some studies describe it as “whistling-face syndrome” [28]. Other features of Sheldon–Hall syndrome include extra skin folds on the neck and short stature. Sheldon–Hall syndrome does not usually affect other organ systems, and intelligence and life expectancy are normal in this disorder. The most affected genes are MYH3, TNNI2, TNNT3, and TPM2. The mode of inheritance is autosomal dominant [33].

Distal arthrogryposis DA5D type. In addition to the features described above, in the 5D type, camptodactyly is more severe (however, the toes usually have a milder presentation). Minor abnormalities include a rounded face, high-arched eyebrows, an upturned nose, and micrognathia. Unilateral or bilateral ptosis is common. The mode of inheritance is autosomal recessive. The most affected gene is ECEL1 [34].

## 2. Materials and Methods

A systematic literature search was performed in PubMed and Embase. All scientific articles available published in the last ten years that matched the search terms “arthrogryposis”, “gene”, or “distal” were screened. After a detailed analysis of the abstracts, we excluded those that were not written in English, summary studies, meta-analyses, experimental descriptions, or descriptions of animal models, as well as articles that were not available through PubMed or Embase. In processing the abstracts, we excluded any articles that did not provide an appropriate answer to our research question.

In our prenatal case, amniocentesis was performed at the 19th gestation week and blood samples from parents were also collected. Our work complies with the principles established in the Declaration of Helsinki. The work was approved by the Ethics Committee of the institute (SE-TUKEB 231). All participants gave written informed consent to participate. Genomic DNA was extracted from amniotic fluid by MN NucleoSpin Tissue XS (Macherey-Nagel, Düren, Germany). In the case of parents, genomic DNA was isolated from blood by an MN NucleoSpin Blood kit (Macherey-Nagel, Düren, Germany). During the extraction process, the general protocol was followed with a minor modification in the elution step (only 50 µL of elution buffer was used). Genomic DNA library from the fetal sample was prepared by using xGen DNA Library Preparation Kit (IDT, Coralville, IA, USA) with 50 ng of DNA input after Qubit 4 fluorimeter (ThermoFisher Scientific, Cleveland, OH, USA) measurement. Coding genomic regions were capture-enriched using an xGen Exome Hybridization Panel v2 (IDT, Coralville, IA, USA) according to the manufacturer’s protocol. For subsequent sequencing, a NovaSeq6000 system (Illumina, San Diego, CA, USA) was used, generating 2 × 150 bp paired-end reads. More than 137 millions reads were generated from a 34 Mb exonic target region with an average depth of sequencing coverage of 181.9x. The obtained sequence reads were aligned to the hg19 human reference sequence and were analyzed using the Genome Analysis Tool Kit (GATK version 4.1.4.1, Broad Institute, Cambridge, MA, USA), and 98.7% of the targeted exome was represented by at least 20-fold coverage. The Franklin by genoox (www.franklin.genoox.com, accessed on 6 December 2023) platform was used to annotate and interpret the identified variants. After the exclusion of low-quality variants, 96,025 alterations were detected. Single-nucleotide substitutions and small insertion and/or deletion variants were identified with our in-house variant-calling pipeline, and exome variant profiles were analyzed with a model of a rare autosomal dominant disorder.

During the variant prioritizing process, all variants outside the exonic and splice regions (±10 variants) were excluded. In the next step, synonymous variants were filtered out. Then, variants with a frequency greater than 5% were excluded. In the next step, ACMG classification by Franklin (www.franklin.genoox.com) was used to keep pathogenic variants, likely pathogenic variants, and variants with unknown significance, and variants which were benign or likely benign in the ClinVar database were excluded. Then, we used the OMIM Morbid genelist and our in-house frequency data to further narrow the list of variants. Finally, the available phenotype and mode of inheritance were used to further narrow the potential findings. After this step, the remaining 6 variants were uniquely evaluated. At this point one remaining variant was excluded because of its adult-onset nature. The four variants classified as VUS based on the ACMG classification criteria and the phenotype–genotype correlation were weaker than the sixth variant, which had pathogenic ACMG classification. So we focused on the sixth variant.

## 3. Results

**Figure 1:**
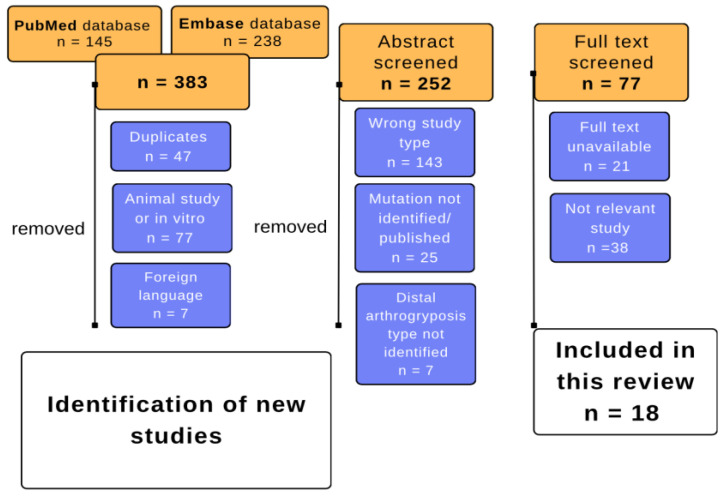
Systematic literature processing.

We obtained 383 hits. In total, 47 duplicates, 77 animal studies, and 7 results written in foreign languages were excluded from the processing. A total of 252 abstracts were reviewed, of which 77 were excluded based on the guidelines detailed above. Of these, 21 studies were excluded because the full text was not available, and in 38 cases, the study was excluded because it was irrelevant to our current systematic review. This process was performed using Rayyan software (Rayyan—a web and mobile app for systemic reviews http://rdcu.be/nzDM, accessed on 6 December 2023) and the flowchart below shows the decision-making process (Figure 1).

Currently, there are 4 studies in preprint, of which 3 describe animal models. Chong et al. present a case of distal arthrogryposis (type not specified) with congenital heart defects, caused by a novel heterogenous missense mutation in ACTC1. These preprinted studies were not included in our systematic review [35].

Since arthrogryposis is a relatively rare and heterogeneous disease, the selection and classification of studies into homogeneous groups is a challenging task. In the described studies, we tried to process cases with detailed case reports and accurate genetic diagnosis (WES/NGS). Since we can mostly rely on case studies, and there are currently no retrospective or prospective studies with many cases of this type, the descriptions of the cases differ depending on the author and study.

A summary table was created from the cases described in the studies processed (Table 3).

From the 18 studies reviewed, 21 cases were summarized. From the studies presented, we included in our table the cases of several patients for whom detailed genetic testing was available. A total of 12 female and 11 male patients were described, with no gender data in 4 cases. In total, 6 cases were diagnosed as DA1, 9 as DA2B, and 12 as DA5D. Prenatally, 9 cases were diagnosed. The abnormality found was homozygous in 9 cases and heterozygous in 18 cases. 

The total number of cases is presented in Table 3.

**Figure 2:**
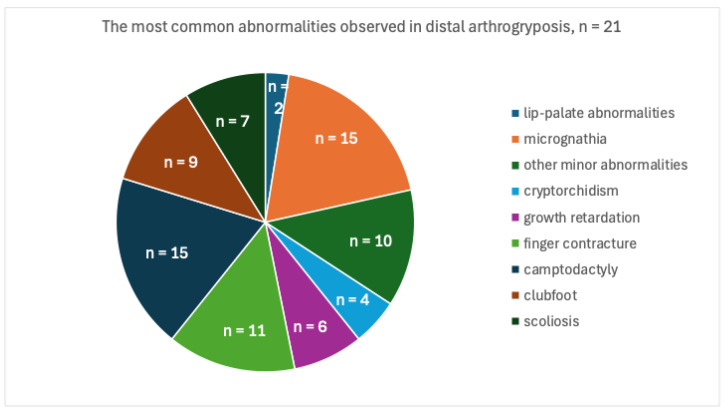
The most common abnormalities observed in distal arthrogryposis.

The observed abnormalities were summarized according to the following criteria: lip-palate abnormalities, micrognathia, other minor abnormalities, cryptorchidism, growth retardation, finger contracture, camptodactyly, syndactyly, clubfoot, and scoliosis. There were no cases of syndactyly among the above, and the distribution of the other abnormalities is shown in Figure 2.

Intelligence was unaffected in all cases, and speech developmental slowness was described in some cases (this may have been due to facial deformities). Motor retardation or delayed motor development may have been due to joint contractures. Breathing difficulties were present in 4 cases. Perinatal exitus did not occur in any case.

### Case Report

In our study, we report a 31-year-old primiparous pregnant woman who was referred to our clinic from an outpatient clinic. During the genetic counseling, positive maternal history was identified with double-side clubfoot and right-hand deformities, and she had surgeries during her infancy. The paternal family history was negative for genetic or inborn disease. During her pregnancy, non-invasive prenatal testing was offered for her, and it was negative for the most frequent trisomies and sex-chromosomal aberrations. At the 19th gestation week during the prenatal ultrasound analysis, clubfoot on both sides and two vessels in the umbilical cord were described. After amniocentesis due to the advanced gestational age and the Hungarian laws, whole-exome sequencing (WES) and karyotyping, which resulted a normal male 46XY genotype, were performed simultaneously.

The fetus had a 46.XY karyotype. Following WES, a known heterozygous pathogenic variant, c.188G>A, p.Arg63His, in the TNNT3 gene was identified in the prenatal sample. This variant is supposed to be pathogenic according to the ACMG classification criteria (PS4, PP3, PM2, PM5, PP5) [54]. Based on Richards et al., classifying PS4 as strong, PM2 and PM5 as moderate, and PP3 and PP5 as supporting cumulatively resulted in a ’pathogenic’ ACMG classification [54]. This variant is not presented in population databases. Computer prediction software consistently assumes adverse effects. Two other variants affected by the same amino acid are also presented in the ClinVar database: a pathogenic/probably pathogenic c.187C>T and a probably pathogenic c.187C>A nucleotide substitution, respectively, resulting in a p.Arg63Cys (Variation ID: 31874) and p.Arg63Ser (Variation ID: 1172549) amino acid substitution. 

**Figure 3:**
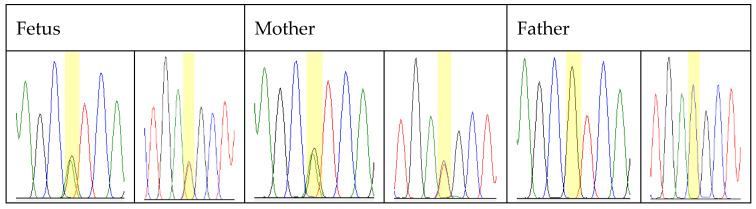
Segregation analysis. Sanger sequencing data of the mutation c.188G>A, p.Arg63His in the TNNT3 gene in a heterozygous state (fetus and mother) and in a normal wild state (father). Green: adenine, red: thymine, blue: cytosine, black: guanine. Yellow highlight: examined position in the sequence.

After the NGS analysis, the result was confirmed by Sanger sequencing in the fetus, revealing the same heterozygous mutation, c.188G>A, p.Arg63His, and targeted sequencing of the parents showed that the affected mother carrying the same heterozygous variant resulted in a C>T heterozygous substitution, while the unaffected father had a wild normal genotype (Figure 3). This result further strengthens the probability of a pathogenic effect of the detected mutation.

The TNNT3 gene encodes the fast skeletal troponin T protein (troponin T3) with an association of distal arthrogryposis type 2B2 with autosomal dominant inheritance, which is characterized by congenital contractures of the distal limb joints and facial dysmorphism. The c.188G>A variant has been reported in the literature in several cases in individuals with distal arthrogryposis [55,56,57,58], and this variant was reported as a de novo occurrence in some of these cases, and it was inherited with the phenotype in a multigenerational family of 18 affected patients [59]. In our case, segregation analysis was performed, and we found that the mother carried the same variant, while the father was homozygous for the wild genotype (Figure 3). Interestingly, in the ultrasound, prominent clubfoot was identified in the mother, who also carried the same mutation and had undergone surgeries to correct the clubfoot, but facial dysmorphism was not detected. 

During the pregnancy, further ultrasound analysis was performed. On the ultrasound scan at 22–23 weeks, the long bones of the limbs were shown to be complete. The hands were in a clenched fist position on both sides throughout the examination. The phalanges appear to be shown completely, with the second and fifth fingers of the right hand in the extended position. On the left hand, the fingers were seen to be congested. The left foot was in the pes equinovarus position. The right ankle was fixed, but no typical pes equinovarus position was seen throughout the study. The umbilical cord contained two blood vessels.

**Figure 4:**
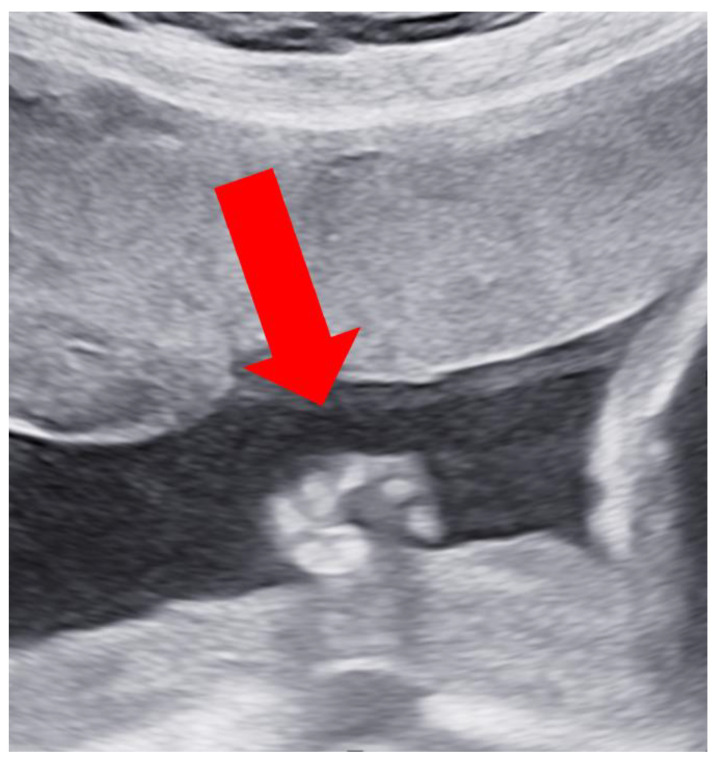
Clenched hand. Transabdominal ultrasound.

**Figure 5:**
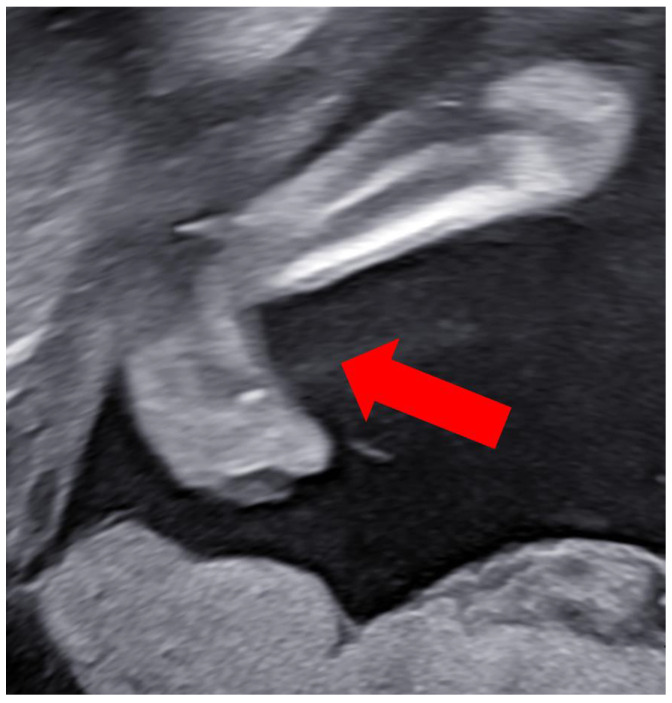
Clubfoot on the left side. Transabdominal ultrasound.

At 27 weeks, the ultrasound showed the long bones of the limbs to be complete. The right hand was clenched in a fist, and finger movement was noted. The left hand clenched in a fist, but no finger movement was discernible (Figure 4). The left foot was held in an equinovarus position (Figure 5). The right ankle was fixed. There was no subcutaneous edema. The umbilical cord contained two blood vessels. 

In the MR examination at week 31, the MR showed the limbs as follows: There were No abnormalities in the upper limbs. The legs were crossed, and as far as could be assessed, the left foot was in a pes equinovarus posture, and the right foot was without pathological deviation. 

A cesarean section was performed at 40 weeks. Following the birth, there were signs of multiple arthrogryposis in the lower and upper limbs. A cast splint was applied during orthopedic care to correct the clubfoot.

## 4. Discussion and Conclusions

Common birth defects like congenital contractures severely complicate daily activity and cause an economic burden to both the patient’s family and the insurance system. Distal arthrogryposis is one of those disorders in which patients show signs of congenital contractures in the distal parts of the body, making normal life extremely difficult [26]. This is the reason why DA requires early multidisciplinary care soon after birth that is well-prepared and adapted to the specific syndrome and potential output [60]. The etiology is thought to be mostly genetic, but in several cases, the molecular etiology of arthrogryposis and the potential biological pathways remain unknown [27]. It is important to highlight that even cases where genetic causes annotbe identified, the negative genetic results could also be informative for clinicians and genetic counselors.

In the family assessed in our case study, a heterozygous pathogenic c.188G>A, p.Arg63His variant in the TNNT3 gene was identified. Our summary of previous DA-associated case reports (Table 3) highlights the critical role of arginine at position 63 of the troponin T3 protein. A change in the arginine at this protein position disrupts the connection with tropomyosin, which results in an increase in calcium ion levels in the skeletal muscles and their contractility, which may eventually cause the development of contractures and limb deformities [22].

Interestingly, in our case, prominent clubfoot was identified in the ultrasound and the mother, who also carried the same TNNT3 p.Arg63His mutation and had undergone surgeries to correct the clubfoot, but facial dysmorphism was not detected. This mutation was previously described in three individuals of a family with DA2B [61]; however, an affected infant with bilateral clubfoot and hand contractures without apparent facial contractures or scoliosis harbored the de novo p.Arg63His variant [40]. However, it is admittedly difficult to diagnose mild forms of facial weakness, particularly in infancy, and thus, it may be difficult to clearly distinguish patients with DA1 (affecting only the hands and feet) from other types of DA [56]. Moreover, Laquérriere et al. in 2014 diagnosed a fetus at 20 weeks of gestation with distal arthrogryposis multiplex congenita with no other features [57]. Thus, it appears that the recurrent TNNT3 p.Arg63His mutation may cause both DA1 and DA2B, suggesting the existence of additional modifying factors in the background of different phenotypes. Even within a single family, highly variable expression can be detected with a wide range of the phenotypic spectrum from an almost nonpenetrant type to a milder form with reduced penetrance and the absence of specific symptoms, such as facial dysmorphism specific to DA1, to a mild DA2B diagnosis with severe hip involvement with subtle facial dysmorphism [59]. 

In their paper published in 2003, Sung et al. investigated the TNNT3 mutation in families with the DA2A or DA2B phenotype [55]. During their investigation, they found a G-A missense mutation in the TNNT3 gene, at position 188 of exon 9, which results in an arginine–histidine amino acid exchange. The investigated missense mutation was also present in other affected members of the family. In the case presented by us, in addition to the known pathogenic mutation reported by Sung et al., both the fetus and the mother carried the heterozygous c.187C>T mutation, which is probably a pathogenic mutation. In the case report of a family living with the c.188G> missense mutation published by Sung et al., there was no detailed phenotype description or follow-up, so a comparison is not possible. Also, we confirmed the dominant inheritance pattern of the c.188G>A mutation, and the dominant inheritance of the newly described c.187C>T mutation can also be assumed. Further family tree research would be necessary to establish a more reliable genetic diagnosis; however, in the case we have described, no further testable living ancestor was available, so further research is required [55].

The described TNNT3 gene is located on the 15.5 locus of chromosome 11 and was confirmed to be a pathogenic mutation in the background of distal-type arthrogryposis 2B. The gene itself was first described by Mao et al. in 1996 [62]. The TNNT3 gene encodes the troponin T protein, which participates in the contraction of striated muscle as part of the troponin complex in the sarcomere. As a result of low calcium levels, the troponin complex inhibits the formation of the connection between thick and thin filaments, and thus, muscle contraction. Due to the mutation in the TNNT3 gene, an afunctional troponin T is formed, so with low calcium levels, the inhibitory effect of the troponin complex does not take effect, and the muscle contraction becomes continuous, resulting in contractures and skeletal malformations [63].

The importance of genetic testing in the presented cases lies in the fact that in both families, the mother had symptoms of distal arthrogryposis, but in their case, no fetal ultrasound results were available for comparison, so it was difficult to assess the fetal symptoms in their pregnancies. However, in light of the genetic analysis, the genotype–phenotype relationship could be confirmed, which facilitates the parents’ decision-making process. As the pregnancy progressed, the parents and clinicians were able to prepare, and immediately after birth, the newborn received treatment appropriate to its genetically confirmed phenotype. This could improve the quality of life of the child and their parents and open up the most effective treatment pathway available in clinical practice.

The main limitation of our manuscript lies in the limitations of the NGS method, as copy number variations could not be identified. As karyotyping was performed in the case of fetus chromosomal level structural changes, balanced translocations could be detected; however, the smaller insertion and deletion outside the NGS resolution remain invisible. Furthermore, other genetic and epigenetic factors, such as uniparental disomy, promoter inactivation, or transcriptional attenuation, could not be detected. To further evaluate the underlying mechanism of phenotypic variability, extended genetic and epigenetic testing is.

The distinction of the boundaries between the different subtypes of DA via phenotyping is hard to achieve, but has been used to aid in the management, counseling, and investigation of the etiology of these genetically and phenotypically related conditions. The example of these families shows that prenatal genetic testing has a role not only in terminating a pregnancy but also in assigning a genotype to a phenotype detected by an ultrasound scan, giving parents all the information currently available to support their decision regarding the termination or continuation of pregnancy and to prepare the couple and their doctors for the necessary interventions. Also, for subsequent pregnancies, knowing the exact genotype of the phenotype will enable the couple to make a decision. Furthermore, in light of the genetic diagnosis close, a neonatal follow-up is required alongside the convening of a multidisciplinary team comprising a neonatologist, pediatric neurologist, pediatric orthopedist, and surgeon specialist. Based on the available ultrasound findings and the genetic diagnosis, they could be prepare well in advance for the treatment after the delivery. In milder cases, contractures may improve with the appropriate orthopedic care and physiotherapy; thus, the preparation and the training of parents is essential to provide the best care at the beginning of the infant’s life [1].

In order to further elucidate the genetic background of DA and to clarify the phenotype–genotype correlation, further genetic studies are warranted with different molecular genetic techniques to increase the genetic resolution. In several cases, with the evolving throughput of ultrasound analysis, longitudinal follow-ups of newborns are needed to develop integrated databases and approaches to predict the combination of different modifying factors and disease-causing variants. Thus, in the future, it is essential to integrate and systematically manage and verify data collected from a number of genetic studies specialized in DA patients. By increasing the available number of validated data, variant classification algorithms and long-term prognostic predictions could be improved to ameliorate the genetic counseling process and provide further information to affected families.

## Figures and Tables

**Table 1:** Mechanism of action of proteins encoded by the most common genes of interest.

Gene Name	Protein Encoded	Locus	Mechanism of Action	Source
TTN	TITIN	2q31.2	Titin helps to form a connection between the thick filament and the Z lineage in the sarcomere. It is also required for the interaction of several sarcomeric proteins.	[14]
CHRNG	ACETYLCHOLINE RECEPTOR	2q37.1	Acetylcholine receptor-dependent mechanisms.	[11]
RYR1	RYANODINE RECEPTOR 1	19q13.2	Cellular calcium homeostasis, redox system.	[15]
ECEL1	ENDOTHELIN-CONVERTING ENZYME-LIKE 1	2q37.1	Membrane-bound metalloprotease and endoprotease, involved in neuropeptide- and peptide-regulated hormone activity. Found in the spine, and lungs, but most highly expressed in the central nervous system.	[16]
ZC4H2	ZINC FINGER C4H2 DOMAIN-CONTAINING PROTEIN	Xq11.2	In a mouse model, the protein is expressed mainly in the hippocampal and dentate processes in the postsynaptic terminal of excitatory neurons.	[17]
NEB	NEBULIN	2q23.3	Nebulin is a large protein of the cytoskeletal matrix. In vertebrae, nebulin is responsible for 3–4% of myofibrillar proteins.	[18]
BICD2	BICD CARGO ADAPTOR 2	9q22.31	Involved in the function of dynein/dynactin in motoneurons.	[19]
CNTNAP1	CONTACTIN-ASSOCIATED PROTEIN 1	17q21.2	Part of the paranodal junction required for myelination of rapidly conducting neurons.	[20]
MAGEL2	MAGE-LIKE 2	15q11.2	Ubiquitin ligase required for endosomal protein recycling.	[21]
TPM2	TROPOMYOSIN 2	9p13.3	Switching the location of the actin–tropomyosin interface between active and relaxed states.	[22]

**Table 3:** Summary of DA case reports for 2019–2024.

Article	Sex	CMA	Prenatally Diagnosed	Gene		Mutation	Mutation Effect	Locus
Zapata-Aldana et al., 2019 [36]	N/A	DA5D	no	PIEZO2	heterozygous	c.8068A>C (p.Ser2690Arg)	LOF	18p11.22-p11.21
Cohen et al., 2023 [37]	female	DA5D	no	ECEL1	homozygous	c.110_155del p.(phe37cysfs * 151)	deletion	2q37.1
Gowda et al., 2021 [38]	male	DA5D	yes	ECEL1	homozygous	c.535A>G (p. Lys179Glu)	missense	2q37.1
Bayram et al., 2016 [27,39]	N/A	DA5D	N/A	ECEL1	homozygous	c.1147C>T; p.Gln383X	missense	2q37.1
	N/A	DA5D	N/A	ECEL1	homozygous	c.2023G>A; p.Ala675Thr	missense	2q37.1
	N/A	DA5D	N/A	ECEL1	homozygous	c.1210C>T; p.Arg404Cys	missense	2q37.1
Pollazzon et al., 2021 [40]	male	DA1	yes	TPM2	heterozygous	c.463G>A, p.(A155T)	missense	9p13.3
	female	DA1	no	TPM2	heterozygous	c.463G>A, p.(A155T)	missense	9p13.3
	female	DA1	N/A	TPM2	heterozygous	c.463G>A, p.(A155T)	missense	9p13.3
	male	DA1	yes	TNNT3	heterozygous	c.187C>T, p.(R63C)	missense	11p15.5
	female	DA1	yes	TNNT3	heterozygous	c.187C>T, p.(R63C)	missense	11p15.5
	female	DA1	yes	TNNT3	heterozygous	c.187C>T, p.(R63C)	missense	11p15.5
	female	DA2B	yes	TNNI2	heterozygous	c.499_501del, p.(E167del)	deletion	11p15.5
	female	DA2B	no	TNNI2	heterozygous	c.499_501del, p.(E167del)	deletion	11p15.5
	male	DA5D	no	ECEL1	heterozygous	c.[1630C>T];[1700C>G], p.[(R544C)];		11p15.5
Wang et al., 2020 [41]	male	DA2B	no	MYH3	heterozygous	c.2506A>G (p.K836E)	missense	17p13.1
	female	DA2B	no	MYH3	heterozygous	c.2506A>G (p.K836E)	missense	17p13.1
	male	DA2B	no	MYH3	heterozygous	c.2506A>G (p.K836E)	missense	17p13.1
Dabaj, Carlier et al. 2022) [42]	male	DA2B	N/A	TNNT3	heterozygous	c.187C>T	missense	11p15.5
	female	DA2B	no	TNNT3	heterozygous	c.187C>T	missense	11p15.5
	female	DA2B	no	TNNT3	heterozygous	c.187C>T	missense	11p15.5
	male	DA2B	no	TNNI2	heterozygous	c.525G>T: p.K175N	missense	11p15.5
Serra et al., 2022 [43]	male	DA5D	second trimester	PIEZO2	heterozygous	c.8181_8183delAGA	GOF	18p11.22-p11.21
Zhang et al., 2020 [44]	male	DA5D	no	ECEL1	homozygous	c.69C>A, p.C23	missense	2q37.1
Li et al., 2022 [45]	male	DA5D	no	ECEL1	homozygous	c.1507-9G>A	missense	2q37.1
Alesi et al., 2021 [46]	female	DA5D	decreased fetal movement	ECEL1	homozygous	c.1507-9G>A	missense	2q37.1
	female	DA5D	decreased fetal movement	ECEL1	homozygous	c.1507-9G>A	missense	2q37.1
Sandaradura et al., 2018 [47]	male	NM-DA	yes	TNNT3	homozygous	c.681+1G>A	missense	11p15.5
Li et al., 2015 [48]	N/A	DA2	N/A	MYBPC1	N/A	c.1075G>A	missense	12q23.2
	N/A	DA2	N/A	MYBPC1	N/A	c.956C>T	missense	12q23.2
Baumann et al., 2017 [49]	male	NM-DA	decreased fetal movement	SYNE1	homozygous	c.26236C>T	premature stop	6q25.2
Ali et al., 2017 [50]	male	DA2B	no	MYH3	heterozygous	c.2015G>A	missense	17p13.1
Iyer et al., 2024 [51]	N/A	DA1	yes	MYBPC1	heterozygous	c.2486_2492del	deletion	12q23.2
Jung et al., 2024 [52]	male	DA5D	yes	ECEl1	heterozygous	c.110_155del	deletion (frameshift)	2q37.1
Neissi et al., 2022 [53]	male	DA1	N/A	TPM2	N/A	c.456G>C	missense	9p13.3

## Data Availability

Not Applicable.
